# Corticospinal tract damage on baseline CT predicts motor recovery and functional outcome in intracerebral haemorrhage

**DOI:** 10.1177/23969873251332769

**Published:** 2025-04-18

**Authors:** Olivia N Murray, Sacha Chiuta, Paul Ryu, Daniel F Hanley, Hiren C Patel, George Harston, Timothy Cootes, Ulrike Hammerbeck, Adrian R Parry-Jones

**Affiliations:** 1 Division of Informatics, Imaging and Data Science, University of Manchester, Manchester, UK; 2 School of Medicinal Sciences, University of Manchester, Manchester, UK; 3 Brain Injury Outcomes, Johns Hopkins Medical Institutions, Baltimore, MD, USA; 4 Department of Neurology, Johns Hopkins University School of Medicine, Baltimore, MD, USA; 5 Salford Royal NHS Foundation Trust, Manchester, UK; 6 Brainomix Ltd, Oxford, UK; 7 Geoffrey Jefferson Brain Research Centre, Manchester Academic Health Science Centre, Northern Care Alliance & University of Manchester, Manchester, UK; 8 Centre of Human and Applied Physiological Sciences, King’s College London, London, UK

**Keywords:** Intracerebral haemorrhage, corticospinal tract, motor recovery, CT, prognosis

## Abstract

**Introduction:**

Corticospinal tract (CST) integrity can predict motor outcome after stroke but requires specialist investigations not routinely performed after intracerebral haemorrhage (ICH). We investigated the feasibility of identifying the CST on routine clinical CT scans, and whether classification of CST overlap with haematoma is associated with motor recovery after ICH.

**Patients and methods:**

An expert observer, blinded to outcome, manually segmented the CST at the posterior limb of the internal capsule (PLIC) and corona radiata (CR) on diagnostic CT scans from 98 randomly selected MISTIE-III trial participants and determined whether CST overlapped with the haematoma. Multivariable linear regression tested for associations between haematoma CST overlap and the motor component of the National Institutes of Health Stroke Scale (baseline & Day 180, rate of recovery), patient reported motor impairment (Stroke Impact Scale [SIS] domain 1) and activity limitation (SIS domains 6&7) at Day 180, and modified Rankin Scale (mRS) at day 180. Three further readers analysed the same scans and the interobserver variability was assessed.

**Results:**

Haematoma and CST overlap occurred exclusively in the CR in 6%, the PLIC in 14% and in both in 52% of patients. CR involvement alone was associated with activity limitation on Day 180. Involvement at the PLIC alone or both the PLIC and CR was independently associated with worse motor outcomes (except rate of recovery, where only involvement of both was associated). Although haematoma and CST overlap remained associated with outcome for other readers, the strength of the association decreased with less expertise, and interobserver kappa scores (κ = 0.47 for CR and κ = 0.45 for PLIC) demonstrated only moderate agreement.

**Discussion and conclusion:**

Haematoma and CST overlap at the level of the PLIC identified on routine CT scans is independently associated with poor motor outcomes, representing a novel prognostic factor. Given moderate interobserver agreement, a more reliable machine-learning classification would be desirable.

## Graphical abstract

**Graphical abstract:**
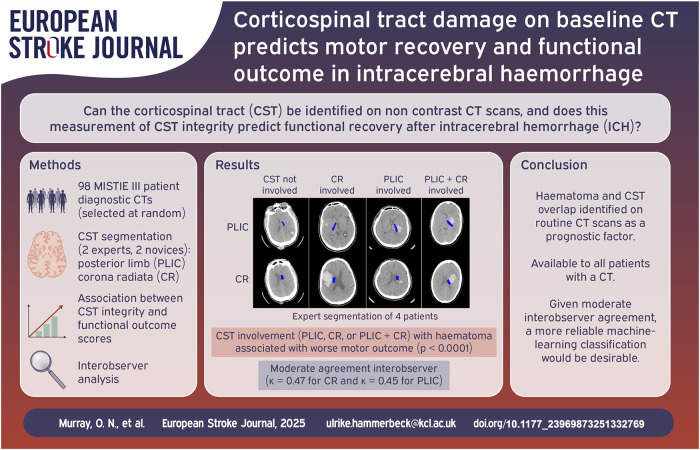

## Introduction

Spontaneous intracerebral haemorrhage (ICH) accounts for 29% of incident strokes globally, but is responsible for nearly half of stroke morbidity and mortality.^1^ Haematoma evacuation after ICH may save lives by reducing mass effect and lowering intracranial pressure,^2^ but it is unclear whether it can improve recovery in survivors. A recent meta-analysis of clinical trials of supratentorial haematoma evacuation demonstrates overall improvement in functional outcome, but when analysis was limited to high quality trials with less risk of bias, there was no clear evidence of overall benefit.^3–6^ The recent ENRICH trial has shown benefit for minimally invasive lobar haematoma evacuation within 24 h of onset.^7^ Therefore, ICH location and the structures involved in the haematoma may be critical in determining functional outcome.

Corticospinal tract (CST) integrity is an important prognostic factor of functional recovery in stroke,^8,9^ but its role in ICH has been less studied. The CST connects the primary motor cortex to the spinal cord, traversing the corona radiata (CR) and the internal capsule, regions frequently involved with haematomas caused by hypertensive arteriolosclerosis.^10^ Identifying patients with irreversible damage to the CST in the hyperacute phase may identify a population with a low probability of good functional outcome that may not benefit by haematoma evacuation.

Functional CST integrity can be probed with single pulse transcranial magnetic stimulation (TMS) and structural integrity by MRI diffusion tensor imaging (DTI-MRI), ^9,11^ though these are challenging in severe ICH and are not routinely used in clinical settings.^12^ Often, the only imaging ICH patients receive is the initial diagnostic non-contrast CT scan. We propose that the CST can be identified on diagnostic CT in regions where it is bordered by contrasting grey matter. In addition, we wanted to establish CST integrity more cephalad, in the CR where tract spread is more diffuse, and is less clearly bordered by contrasting tissue.

This study aimed to develop a reproducible standardised method for manually identifying the location of the CST at the level of the PLIC and CR in acute, diagnostic, non-contrast CT scans, and assess the overlap between the haematoma and CST. We then sought to establish if CST involvement with the haematoma was an independent predictor of worse baseline motor deficit, motor recovery and motor outcome, providing a practical tool for prognostication in ICH patients.

## Patients and methods

### Participants

To test for associations between CST integrity and motor outcomes, we used diagnostic CT scans and clinical data from the minimally invasive surgery with thrombolysis in intracerebral haemorrhage evacuation (MISTIE III) clinical trial.^6^ MISTIE III was an international, multicentre, randomised controlled trial of minimally invasive catheter evacuation followed by thrombolysis versus standard medical care in patients with spontaneous, non-traumatic, supratentorial ICH (>30 ml), collected between December 30, 2013 and August 15, 2017. After a sample size calculation assuming a Cohen’s kappa value of 0.8, minimum detectable difference of 0.1 and a lower confidence bound of 0.7, baseline non-contrast CT scans and longitudinal clinical data were extracted for 98 participants selected at random from the entire trial population. All observers were blinded to clinical data. For development of the standard operating procedure (SOP) for CST segmentation, 10 diagnostic CT scans within 3 h of onset of ICH were randomly selected from a prior, observational cohort study (NHS REC Ref: 15/NW/0387).

### Clinical measures and outcomes

Motor impairment was measured as the sum of the arm, leg and face scores of the National Institutes of Health Stroke Scale (NIHSS; NIHSS questions 4, 5 and 6; range 0–19), which we will subsequently refer to as ‘motor NIHSS’, with higher scores indicating greater motor deficit. NIHSS scores were recorded in the MISTIE III trial at baseline and days 7, 30, 180 and 365. The patient reported Stroke Impact Scale (SIS) was used to measure patient reported motor impairment (‘physical problems’ domain 1) and patient reported activity limitations (mobility and hand function domains 6 and 7).^13^ The scores for each SIS domain were standardised to a 0–100 scale, as is standard for analysis of the SIS.^14^

As we were interested in the effect of CST integrity on long term functional outcome, we chose day 180 motor NIHSS score as our primary outcome. Secondary outcomes included baseline motor NIHSS score, the rate of motor recovery to day 365, SIS impairment and activity limitation at day 180 and modified Rankin Scale (mRS) at day 180. Rate of motor recovery was derived by extracting the gradient of the line of best fit to a plot of the motor NIHSS score at the natural logarithm of 5 timepoints (baseline, days 7, 30, 180 and 365).

### CST segmentation

A SOP was iteratively developed for segmenting the PLIC and CR and assessing haematoma overlap, as described in the Supplemental Materials. The SOP instructed observers to outline the ipsilesional PLIC on all CT slices where the thalamus and lentiform nucleus were clearly visible, using the border between grey and white matter to mark the PLIC border. To identify the CST at the CR, the most rostral slice on which the septum pellucidum could be identified was selected, and on this the ipsilesional lateral ventricle was divided into thirds along the anterior-posterior axis and a rectangle measuring 1 cm in the medio-lateral axis was annotated next to the middle third of the lateral ventricle and parallel to its lateral wall. Observers classified whether the haematoma overlapped the CST at the level of the PLIC or CR as ’not involved’, ’partially involved’ or ’completely involved’.

Four observers segmented the PLIC and CR on CTs from the MISTIE-III dataset in the axial plane, following the SOP, using the medical imaging software OsiriX (Pixmeo SARL, Switzerland). The primary observer (APJ) was a vascular neurologist with over 20 years of experience in the care of ICH patients. To establish interobserver repeatability in less experienced observers, two observers (PR and SC) segmented all scans. One had 2 years’ experience as a Clinical Data Programmer Analyst at the Brain Injury Outcomes Neuroimaging Center, Johns Hopkins University; the other was a senior medical student at the University of Manchester completing a 6-month stroke research project. To determine whether differences between expert and less experienced readers were due to the SOP or observer experience, another expert (HP), a neurovascular surgeon with over 20 years of experience, segmented 42 scans. All observers were blinded to clinical outcomes.

### Statistical analysis

CST involvement was categorised as one of four levels; no involvement of the CST, (denoted as CST_spared_), involvement of the CR, involvement of the PLIC and involvement of both the PLIC and CR. Involvement included both ‘partially involved’ or ‘completely involved’. For baseline tables and result visualisations, all patients with any CST involvement (CR, PLIC or both) were grouped into CST_involved_. Patients missing data at any timepoint were not included in the analysis of that timepoint. Data missingness is described in Supplemental Materials Figure 1.

To investigate any differences between CST_involved_ and CST_spared_ groups at baseline, baseline characteristics for each group were compared using two tailed t-tests. The mean motor NIHSS at each timepoint (baseline, day 7, 30, 180, 365) were compared for CST_involved_ and CST_spared_ groups with two-tailed t-tests, and visualised as a violin plot.

The association between CST involvement in the haematoma (as identified by expert observer A) and motor outcomes was investigated using multivariable linear regression analyses with the dependent variables as baseline motor NIHSS, day 180 motor NIHSS, SIS impairment, SIS function and mRS, and rate of motor recovery to day 365. Here, we consider all four levels of CST involvement separately (no involvement, CR involved, PLIC involved, PLIC and CR involved) to investigate the effect of involvement at different locations along the CST on outcome. Age, sex, natural logarithm of ICH volume, intraventricular haemorrhage (IVH) volume, baseline motor NIHSS score and randomisation to surgical or medical group were all controlled for as covariables, based on prior clinical and research knowledge.^15^

### Interobserver analysis

Interobserver variability was conducted with pairwise comparisons of each observer’s segmentations using the mean Dice Similarity Coefficient (DSC). To assess the interobserver agreement on the overlap of haematoma with the PLIC and CR, Fleiss’ kappa scores were calculated for each structure. To assess the impact of overlap between the haemorrhage and PLIC on pairwise DSC, a one-way ANOVA test was performed between; ’not involved’, ’partially involved’ and ’completely involved’ groups as defined by observer APJ.

The linear regression analyses were repeated for the classification of CST involvement of the less experienced observers PR and SC, with the same outcome variables and covariables as used for observer APJ.

## Results

### Baseline characteristics

**Figure 1.**
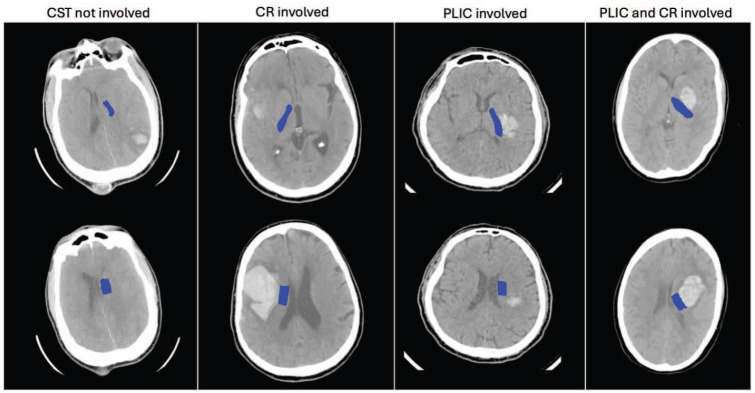
An example of PLIC (top row) and CR (bottom row) segmentations (blue) for four patients with different levels of CST involvement as defined by observer A.

Baseline characteristics of the 98 MISTIE-III patients included in the analysis are shown in Table 1, grouped by whether the CST was involved (denoted as CST_involved_; n = 71) or not (denoted as CST_spared_; n = 27). PLIC and CR segmentations are shown in Figure 1, for three patients with different levels of involvement. CST_involved_ patients were significantly younger, had predominantly deep ICH (89%), had more IVH (46%), lower GCS, and higher NIHSS. By contrast, CST_spared_ patients had predominantly lobar ICH location (85%), less IVH (3%), higher GCS, and lower NIHSS. There were no significant differences between sex, ethnicity and haematoma volume. The motor component of NIHSS at baseline was significantly greater for CST_involved_ patients than the median score for CST_spared_ patients (11 vs 7).

**Table 1.** Baseline characteristics.

Characteristic	Level	CST_involved_	CST_spared_	CST_spared_ vs CST_involved_
		(*N* = 71)	(*N* = 27)	(*p*-Value)
Age (years)	Mean (SD)	59.1 (12.9)	67.9 (9.7)	0.0017
	Minimum-maximum	31–80	39–80	
Sex				1^†^
Male	*N* (%)	40 (56%)	15 (56%)	
Female	*N* (%)	31 (44%)	12 (44%)	
Ethnicity				0.53^†^
White	*N* (%)	42 (59%)	17 (63%)	
White Hispanic	*N* (%)	7 (10%)	1 (4%)	
African American		17 (24%)	7 (26%)	
Asian		4 (6%)	1 (4%)	
Native American		1 (1%)	0	
African American and White		0	1 (4%)	
Pre-randomisation GCS	Median (IQR)	9 (8–12.5)	13 (10–14)	0.012
	Minimum-Maximum	7–15	6–15	
GCS classification				0.04^†^
Mild (14–15)	*N* (%)	10 (14%)	9 (33%)	
Moderate (9–13)	*N* (%)	37 (52%)	14 (52%)	
Severe (3–8)	*N* (%)	24 (34%)	4 (15%)	
NIHSS on admission/baseline	Median (IQR)	22 (18.5–26)	16 (10–20)	<0.0001
	Minimum-Maximum	11–39	2–33	
NIHSS on admission/baseline				0.0004^†^
Minor (1–4)	*N* (%)	0 (0%)	1 (4%)	
Moderate (5–15)	*N* (%)	8 (11%)	12 (44%)	
Moderate-severe (16–20)	*N* (%)	23 (32%)	8 (30%)	
Severe (21–40)	*N* (%)	40 (56%)	6 (22%)	
Sum of NIHSS motor domains (4–6)	Median (IQR)	11(9–12.5)	8(4–10)	<0.0001
	Minimum-Maximum	6–19	0–19	
Baseline ICH volume (mL)	Median (IQR)	36 (26.5–50.2)	38.6 (30–50.6)	0.62
	Minimum-Maximum	7.9–97.4	19.2–71.1	
Intraventricular haemorrhage	*N* (%)	33 (46%)	10 (37%)	0.54^†^
Baseline IVH volume (mL)	Mean (SD)	3.1 (9.0)	2.65 (7.0)	0.81
	Minimum-maximum	0–67.7	0–33.1	
Haematoma location				<0.0001^†^
Deep	*N* (%)	63 (89%)	4 (15%)	
Lobar	*N* (%)	8 (11%)	23 (85%)	
Randomisation				0.06^†^
Surgical	*N* (%)	38 (54%)	8 (30%)	
Medical	*N* (%)	33 46%)	19 (70%)	

*T*-tests were used for comparison unless denoted by † in which case chi-squared tests were used.

### Association between CST involvement and outcome

**Figure 2.**
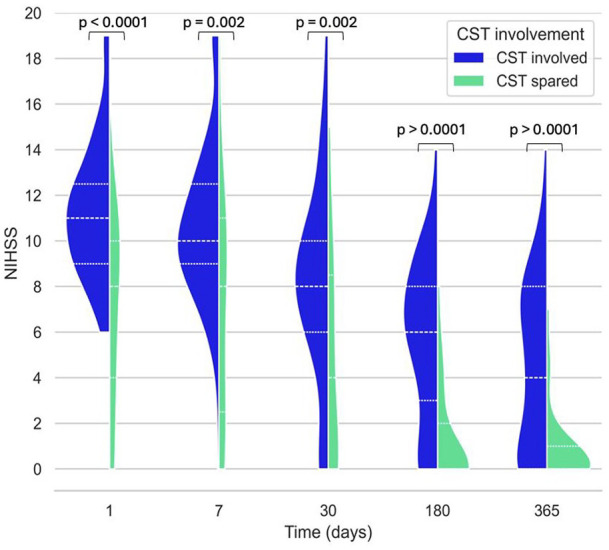
A violin plot comparing motor NIHSS scores for CST_involved_ (CR, PLIC or both) versus CST_spared_ at 1, 7, 30, 180 and 365 days post stroke. Groups are compared at each timepoint with a Mann-Whitney *U* test.

Figure 2 shows motor NIHSS for CST_involved_ and CST_spared_ groups at different timepoints. The mean motor NIHSS for CST_involved_ was significantly higher than for CST_spared_ at baseline, day 7, 30, 180 and 365.

### Association between baseline characteristics and outcomes

Multiple linear regression models (Table 2) indicated that involvement of the CST at the PLIC, or both the CR and PLIC, was independently associated with higher motor NIHSS at day 180 and at baseline. The involvement of both the PLIC and the CR was independently associated with a slower rate of improvement in the NIHSS to day 365. For motor NIHSS at day 180, older age, higher ICH volume and higher baseline motor NIHSS remained independently associated with worse outcome. For baseline motor NIHSS, no other variables in the model were significantly associated with outcome. For rate of improvement of the motor NIHSS to day 365, older age and randomisation to surgery were associated with slower improvement of the motor NIHSS to day 365, but higher baseline motor NIHSS was independently associated with a faster rate of improvement.

**Table 2.** Association between corticospinal tract involvement by expert observer and observed motor impairment. Results of multivariate linear regression models to determine associations between CST involvement classification by expert observer and motor NIHSS at baseline, day 180 and the rate of change from baseline to day 365. Beta coefficients are given with 95% CI.

Covariate	Sum of NIHSS motor domains (4 to 6)					
	Baseline		Day 180		Rate of change to Day 365 [NIHSS/ln(days)]	
	β coefficient (95% CI)	*p*	β coefficient (95% CI)	*p*	β coefficient (95% CI)	*p*
Corticospinal tract involvement						
CR	2.84 (−0.49 to 6.16)	0.09	1.95 (−0.61 to 4.51)	0.13	0.12 (−0.40 to 0.65)	0.64
PLIC	4.62 (1.99 to 7.26)	0.0008	3.16 (1.11 to 5.21)	0.003	0.39 (−0.02 to 0.79)	0.06
Both	4.08 (2.35 to 5.81)	<0.0001	4.68 (3.27 to 6.11)	<0.0001	0.70 (0.43 to 0.98)	<0.0001
Not involved	Ref		Ref		Ref	
Baseline NIHSS motor domains (Sum 4–6)	N/A	N/A	0.28 (0.13 to 0.44)	0.0006	−0.11 (−0.15 to −0.09)	<0.0001
Age (years)	−0.03 (−0.10 to 0.029)	0.3	0.06 (0.02 to 0.11)	0.006	0.02 (0.01 to 0.02)	0.0003
Sex (Male)	0.07 (−1.44 to 1.57)	0.93	−0.55 (−1.61 to 0.52)	0.31	−0.14 (−0.35 to 0.06)	0.16
IVH (mL)	0.07 (−0.01 to 0.16)	0.088	0.04 (−0.02 to 0.09)	0.2	0.01 (−0.00 to 0.02)	0.11
ICH volume* (mL)	1.74 (−0.026 to 3.50)	0.053	1.33 (0.03 to 2.65)	0.046	0.16 (−0.07 to 0.39)	0.17
Intervention group (surgery)	N/A	N/A	0.83 (−0.19 to 1.87)	0.11	0.25 (0.05 to 0.44)	0.014

* ICH volumes were transformed to natural logarithm to achieve normal distribution prior to analysis.

Multiple linear regression models (Table 3) indicated that involvement of the CST at the PLIC alone, or both the CR and PLIC, was independently associated with worse patient reported outcome measures of impairment (SIS domain 1) and greater limitations in patient reported activity (SIS domains 6&7) at day 180. In addition, involvement of the CR alone was associated with worse scores in SIS domain 1 but not domains 6&7. In both multiple regression models, both older age and higher baseline NIHSS were associated with worse outcomes. For SIS domain 1, male sex remained independently associated with better outcomes. Involvement of the CST at the PLIC alone, and both the CR and PLIC, were independently associated with worse mRS at day 180, along with higher baseline motor NIHSS, older age and increased haematoma volume.

**Table 3.** Association between corticospinal tract involvement by expert observer and patient reported motor impairment and activity limitation.

Covariate	SIS domain 1 at day 180 (Motor impairment)		SIS domains 6&7 at day 180 (Activity limitation)		mRS at day 180	
	β coefficient (95% CI)	*p*	β coefficient (95% CI)	*p*	β coefficient (95% CI)	*p*
CST involvement						
CR	−16.73 (−39.30 to 5.83)	0.14	−55.19 (−103 to −7.37)	0.024	0.05 (−1.09 to 1.192)	0.93
PLIC	−41.18 (−60.54 to −21.83)	<0.0001	−95.27 (−136.3 to −54.26)	<0.0001	1.41 (0.46 to 2.36)	0.004
Both	−36.86 (−50.17 to −23.55)	<0.0001	−76.99 (−105.2 to −48.78)	<0.0001	1.186 (0.53 to 1.84)	0.0005
Not involved	Ref		Ref		Ref	Ref
Baseline NIHSS motor domains (sum 4–6)	−3.00 (−4.50 to −1.49)	0.0002	−5.39 (−8.59 to −2.20)	0.0012	0.11 (0.03 to 0.18)	0.004
Age (years)	−0.57 (−0.98 to −0.15)	0.0079	−1.76 (−2.63 to −0.88)	0.0002	0.04 (0.02 to 0.06)	0.0004
Sex (Male)	12.44 (2.23 to 22.65)	0.018	10.91 (−10.72 to 32.54)	0.32	−0.22 (−0.72 to 0.29)	0.39
IVH (mL)	0.15 (−0.38 to 0.69)	0.57	0.34 (−0.79 to 1.48)	0.55	−0.01 (−0.04 to 0.02)	0.37
ICH volume* (mL)	−2.17 (−14.7 to 10.36)	0.73	−21.46 (−48.01 to 5.10)	0.11	0.76 (0.15 to 1.37)	0.02
Randomisation to surgery	−6.87 (−16.76 to 3.03)	0.17	−19.23 (−40.20 to 1.74)	0.072	−0.21 (−0.71 to 0.28)	0.39

Results of multivariate linear regression models to determine associations between CST involvement classification by expert observer and patient reported motor impairment (Stroke Impact Scale [SIS] domain 1) and activity limitations relevant to gait and upper limb function (SIS domains 6 & 7) at day 180. Beta coefficients are given with 95% CI.

* ICH volumes were transformed to natural logarithm to achieve normal distribution prior to analysis.

### Interobserver analysis

The pairwise mean DSC are shown in Table 4.

**Table 4.** Mean pairwise DSC for pairs of observers.

Observer	APJ	PR	SC	HP
APJ		59%	47%	52%
PR			44%	45%
SC				50%
HP				

The Fleiss’ kappa scores for involvement of PLIC and CR in the ICH were 0.45 and 0.47 respectively. These scores reflect moderate agreement (0.41–0.60) for both anatomical locations. A one-way ANOVA test performed on the DSCs in each of the three involvement groups found no statistically significant difference between the means of these three groups (*F* = 0.32 and *p* = 0.73).

The associations between involvement classifications and outcome differed when performed by inexperienced observers. Multiple linear regression analysis found for observers PR and SC (Supplemental Materials Tables 1 and 2), all associations (except the effect of ICH and IVH volume for observer SC) remained in the same direction as observer APJ but were generally weaker. The effect of PLIC involvement tended to lose significance when repeated with inexperienced observers, however the significance and effect size of CR involvement was greater in observer SC than observer APJ. Notably, for both inexperienced observers, the significance and effect size of randomisation to surgery (vs medical) on outcome was greater than for the expert observer.

## Discussion

We here demonstrate that overlap between the haematoma and PLIC or both the PLIC and CR, identified on routine clinical non-contrast CT scans after ICH, are strongly and independently associated with greater motor impairment. This association is evident at day 180, baseline, and in patient reported impairment and activity limitation at day 180 and there is also a significant association with worse mRS scores at day 180. Although interobserver agreement in terms of categorisation of involvement and overlap of segmentations was moderate, segmentations by less experienced observers remained significant predictors of motor outcomes.

We have found a robust, independent association between involvement of the PLIC (either alone or in combination with the CR) and poor patient-reported and investigator-measured motor outcomes. However, CR involvement alone was less closely associated with outcome. This may be due to the relative dispersion of the CST at the level of the CR, as it fans out towards the primary motor cortex.^16^ Additionally, CST identification in the CR is less specific due to the lack of a clear grey-white matter border, unlike the PLIC, where the lentiform nucleus and thalamus provide clear lateral borders. For outcomes based on the motor NIHSS, involvement of both the PLIC and the CR was a stronger predictor of poor outcome than PLIC involvement alone, suggesting more extensive CST damage at baseline is occurring when both regions are involved. For patient-reported outcome measures, however, PLIC involvement on its own was a stronger predictor. The cause for this disparity is uncertain.

It is of interest that randomisation to surgery was an independent predictor of a slower rate of recovery of the NIHSS to day 365. The STICH trials suggested a signal of benefit for patients with lobar ICH, that did not reach statistical significance in STICH-II.^5^ More recently, the ENRICH trial has confirmed benefit of minimally invasive surgery in lobar ICH.^7^ Lobar ICH excludes involvement of deep structures, including the PLIC, providing a possible mechanistic explanation for the lack of benefit for patients with deep ICH in ENRICH. However, other trials have not clearly shown a lack of benefit in deep ICH so simply excluding all deep ICH from surgery may be too simplistic an approach.^3^ Ongoing trials are including patients with deep ICH (DIST [NCT05460793], EVACUATE [NCT04434807], EMINENT-ICH [NCT05681988]) and it may be that the key factor dictating whether patients can benefit from minimally invasive surgery for deep ICH is whether the CST is involved at the PLIC. Our method may provide a means to easily distinguish such patients at baseline using routine CT scans.

It is encouraging that the association between CST involvement and motor outcomes remains, even though it is diminished, when the segmentation is performed by less experienced CT scan readers. However, we observe interobserver variation in the spatial overlap of the segmentation. Despite a standardised SOP created to maximise observer agreement, the highest agreement between pairs of observers as measured by the Dice coefficient was 59%, highlighting the challenges of segmenting the CST on plain CT. The complexity of segmenting the CR could also be considered a limitation. The PLIC delineation is more intuitive, as it involves tracing a clear grey-white matter border, and could be more feasible to apply at the bedside. The sample size (*n* = 98) was selected to balance statistical considerations with the time required for four observers to segment the scans, however this limits our power to perform subgroup analyses, including comparisons of different treatment groups.

The advantages of using the baseline CT in ICH to assess CST involvement is that they are a routine part of inpatient care^17^ and provide high contrast differentiation between the haematoma and surrounding brain tissue. Although TMS and DTI-MRI can establish the integrity of the CST ^18,19^ these are costly and challenging in ICH, where stroke severity and reduced consciousness are often greater than seen in ischaemic stroke.^20^ However, using the PLIC as observed on the CT as a surrogate for CST injury introduces uncertainty, as we have no confirmation of structural damage or functional consequences provided by DTI-MRI or TMS. One of the limitations of this study is that we have no objective measure of correctness, and the only way of assessing CST segmentation accuracy is to assess how strongly it predicts motor outcome after stroke, as we know from TMS and DTI studies that if the CST is truly damaged, motor function will be impacted. If we follow this logic, then, the expert observer has managed to produce the most anatomically accurate segmentations of the CST. Further research employing machine learning with DTI-MRI segmentations of the CST as a ground truth may provide a superior alternative.

In conclusion, our standardised approach to assessing corticospinal tract damage on diagnostic CT imaging demonstrates a strong association with measures of motor and functional outcome after stroke, holding promise for prognostication and personalised approaches to rehabilitation. The apparent detrimental effect of surgery on motor outcomes suggests that our approach may identify a subset of patients with an intact corticospinal tract who may be more likely to benefit from surgery, even in deep ICH. The consistent association between CST involvement and poor motor outcome demonstrated by both expert and less experienced observers supports the clinical utility of the method despite the interobserver variability in segmentation overlap. The robustness of the tool may be improved using automated segmentation techniques.

## Supplemental Material

sj-pdf-1-eso-10.1177_23969873251332769 – Supplemental material for Corticospinal tract damage on baseline CT predicts motor recovery and functional outcome in intracerebral haemorrhage Supplemental material, sj-pdf-1-eso-10.1177_23969873251332769 for Corticospinal tract damage on baseline CT predicts motor recovery and functional outcome in intracerebral haemorrhage by Olivia N Murray, Sacha Chiuta, Paul Ryu, Daniel F Hanley, Hiren C Patel, George Harston, Timothy Cootes, Ulrike Hammerbeck and Adrian R Parry-Jones in European Stroke Journal
