# Supplementary material for: Corticospinal tract damage on baseline CT predicts motor recovery and functional outcome in intracerebral haemorrhage
Source: Eur Stroke J. 2025 Apr 18;10(4):1383–91. doi: 10.1177/23969873251332769 (PMC12008156; doi:10.1177/23969873251332769)
Supplement: sj-pdf-1-eso-10.1177_23969873251332769 – Supplemental material for Corticospinal tract damage on baseline CT predicts motor recovery and functional outcome in intracerebral haemorrhage [file sj-pdf-1-eso-10.1177_23969873251332769.pdf]

Supplementary materials

Supplementary tables

Table S1: Baseline characteristics split into 4 levels of CST involvement. This table contains a breakdown of Table 1, with the $CST_{involved}$ group split further into CR involved, PLIC involved and PLIC and CR involved.

Characteristic	Level	$CST_{involved}$ (N=71)			CST_{spared}
		CR<br><br>(N=6)	PLIC<br><br>(N=14)	PLIC and CR<br><br>(N=51)	(N=27)
**Age** (years)	Mean (SD)<br>Minimum-Maximum	63.83 (7.8)<br>53-73	51.14 (15.8)<br>31-80	60.69 (11.8)<br>41-80	67.9 (9.7)<br>39-80
**Sex**<br>Male<br>Female	 N (%)<br>N (%)	 6 (100%)<br>0	 6 (43%)<br>8 (57%)	 28 (55%)<br>23 (45%)	 15(56%)<br>12 (44%)
**Ethnicity**<br>White<br>White Hispanic<br>African-American<br>Asian<br>Native American<br>African-American and White	 N (%)<br>N (%)<br>N (%)<br>N (%)<br>N (%)<br>N (%)	 3 (50%)<br>1 (17%)<br>1 (17%)<br>1 (17%)	 7 (50%)<br>3 (21%)<br>3 (21%)<br>1 (7%)	 32 (63%)<br>3 (6%)<br>13 (25%)<br>2 (4%)	 17 (63%)<br>1 (4%)<br>7 (26%)<br>1 (4%)<br>0<br>1(4%)
**Pre-randomisation Glasgow Coma Scale score**	Median (IQR)<br>Minimum-Maximum	9 (8.25-9)<br>7-14	11 (8.25-13.75)<br>7-14	9 (8.25-12)<br>7-15	13 (10-14)<br>6-15
**Glasgow Coma Score Classification**<br>Mild (14-15)<br>Moderate (9-13)<br>Severe (3-8)	 N (%)<br>N (%)<br>N (%)	 1 (17%)<br>3 (50%)<br>2 (33%)	 4 (29%)<br>7 (50%)<br>3 (21%)	 5 (10%)<br>27 (53%)<br>19 (18%)	 9 (33%)<br>14 (52%)<br>4 (15%)
**NIHSS on admission/baseline**	Median (IQR)<br>Minimum-Maximum	20.5 (16-24.25)<br>11-26	20 (17.5-24)<br>15-36	22 (19-26)<br>14-39	16 (10-20)<br>2-33
**NIHSS on admission/baseline**<br>Minor (1-4)<br>Moderate (5-15)<br>Moderate-severe (16-20)	 N (%)<br>N (%)<br>N (%)	 0<br>2 (33%)<br>1 (17%)	 0<br>1 (7%)<br>7 (50%)	 0<br>5 (10%)<br>15 (29%)	 1 (4%)<br>12 (44%)<br>8 (30%)

Severe (21-40)	N (%)	3 (50%)	6 (43%)	31 (61%)	6 (22%)
**Sum of NIHSS motor domains (4 to 6)**	Median (IQR)<br>Minimum-Maximum	11.5 (11-12)<br>8-12	11.5 (9.25-12.75)<br>7-19	11 (9-13.5)<br>6-19	8 (4-10)<br>0-19
**Baseline ICH Volume** (mL)	Median (IQR)<br>Minimum-Maximum	60.86 (48.9-68.3)<br>45.1-97.4	29.36 (23.8-37.5)<br>8.86-54.4	35.87 (26.4-48.7)<br>7.9-76.5	38.6 (30-50.6)<br>19.2-71.1
**Intraventricular haemorrhage**	N (%)	3 (50%)	5 (36%)	25 (49%)	10 (3%)
**Baseline IVH Volume** (mL)	Mean (SD)<br>Minimum-Maximum	4.5 (6.2)<br>0-15.3	1.1 (2.8)<br>0-10.1	3.5 (10.3)<br>0-67.7	2.65 (7.0)<br>0-33.1
**Hematoma location**					
Deep	N (%)	1 (17%)	13 (93%)	49 (96%)	4 (15%)
Lobar	N (%)	5 (83%)	1 (7%)	2 (4%)	23 (85%)
**Randomisation**					
Surgical	N (%)	3 (50%)	8 (57%)	27 (53%)	8 (30%)
Medical	N (%)	3 (50%)	6 (43%)	24 (47%)	19 (70%)

Supplementary **Table S2: Association between corticospinal tract involvement by novice observer PR and observed motor impairment.** Results of multifactorial linear regression models to determine associations between corticospinal tract involvement classification by novice observer PR and observed motor impairment (sum of NIHSS motor domains 4 to 6) at baseline, day 180, the rate of change from baseline to day 365, and the proportion of recovery to Day 365. Beta coefficients are given with 95% confidence interval. *ICH volumes were transformed to natural logarithm to achieve normal distribution prior to analysis. IVH, intraventricular hemorrhage; ICH, intracerebral hemorrhage.

	Sum of NIHSS motor domains (4 to 6)					
	Baseline		Day 180		Rate of change to Day 365 [NIHSS/ln(days)]	
	**β coefficient (95% CI)**	***p***	**β coefficient (95% CI)**	***p***	**β coefficient (95% CI)**	***p***
**Corticospinal tract involvement**						
CR	2.16 (-1.42 to 5.74)	0.23	0.77 (-2.35 to 3.89)	0.62	0.07 (-0.65 to 0.79)	0.84
PLIC	0.99 (-1.32 to 3.29)	0.40	0.71 (-1.26 to 2.69)	0.48	0.07 (-0.30 to 0.44)	0.70
Both	3.46 (1.55 to 5.37)	0.0005	2.32 (0.54 to 4.11)	0.012	0.31 (-0.02 to 0.65)	0.06
Not involved	Ref		Ref		Ref	
**Sum of NIHSS motor domains (4 to 6) at baseline**	N/A	N/A	0.41 (0.22 to 0.60)	<0.0001	-0.1 (-0.13 to -0.06)	<0.0001
**Age** (years)	-0.06 (-0.12 to 0.002)	0.056	0.06 (0.01 to 0.11)	0.018	0.02 (0.00 to 0.03)	0.001
**Sex** (Male)	0.52 (-0.95 to 1.98)	0.48	-0.29 (-1.58 to 1.00)	0.66	-0.13 (-0.37 to 0.11)	0.29
**IVH** (ml)	0.07 (-0.01 to 0.16)	0.097	0.03 (-0.04 to 1.01)	041	0.01 (-0.01 to 0.02)	0.27
**ICH volume*** (ml)	1.10 (-0.54 to 2.74)	0.19	1.18 (-0.38 to 2.74)	0.14	0.15 (-0.13 to 0.42)	0.29
**Randomization to surgery**	N/A	N/A	1.29 (0.01 to 2.56)	0.048	0.32 (0.09 to 0.56)	0.007

Supplementary **Table S3: Association between corticospinal tract involvement by novice observer SC and observed motor impairment.** Results of multifactorial linear regression models to determine associations between corticospinal tract involvement classification by novice observer SC and observed motor impairment (sum of NIHSS motor domains 4 to 6) at baseline, day 180, the rate of change from baseline to day 365, and the proportion of recovery to Day 365. Beta coefficients are given with 95% confidence interval. *ICH volumes were transformed to natural logarithm to achieve normal distribution prior to analysis. IVH, intraventricular hemorrhage; ICH, intracerebral hemorrhage.

	Sum of NIHSS motor domains (4 to 6)					
	**Baseline**		**Day 180**		**Rate of change to Day 365 [NIHSS/ln(days)]**	
	**β coefficient (95% CI)**	***p***	**β coefficient (95% CI)**	***p***	**β coefficient (95% CI)**	***p***
**Corticospinal tract involvement**						
CR	2.65 (0.53 to 4.77)	0.015	2.74 (1.04 to 4.44)	0.002	0.43 (0.10 to 0.75)	0.011
PLIC	3.09 (0.62 to 5.56)	0.015	2.70 (0.51 to 4.89)	0.016	0.15 (-0.28 to 0.58)	0.49
Both	2.21 (0.27 to 4.15)	0.026	3.73 (2.21 to 5.25)	<0.0001	0.47 (0.18 to 0.75)	0.0017
Not involved	Ref		Ref		Ref	
**Sum of NIHSS motor domains (4 to 6) at baseline**	N/A	N/A	0.36 (0.19 to 0.53)	<0.0001	-0.10 (-0.13 to -0.07)	<0.0001
**Age** (years)	-0.07 (-0.13 to -0.01)	0.030	0.06 (0.01 to 0.11)	0.015	0.01 (0.01 to 0.02)	0.017
**Sex** (Male)	0.30 (-1.22 to 1.83)	0.70	-0.38 (-1.57 to 0.80)	0.52	-0.11 (-0.33 to 0.12)	0.34
**IVH** (ml)	0.08 (-0.01 to 0.17)	0.094	0.03 (-0.04 to 0.09)	0.44	0.01 (-0.00 to 0.02)	0.23
**ICH volume*** (ml)	0.8 (-0.93 to 2.54)	0.36	-0.22 (-1.68 to 1.24)	0.76	-0.09 (-0.36 to 0.18)	0.50
**Randomization to surgery**	N/A	N/A	1.26 (0.10 to 2.42)	0.034	0.34 (0.12 to 0.56)	0.004

Supplementary Table S4: Association between corticospinal tract involvement by novice observer PR and patient reported motor impairment and activity limitation. Results of multifactorial linear regression models to determine associations between corticospinal tract involvement classification by novice observer PR and patient reported motor impairment (Stroke Impact Scale [SIS] domain 1) and activity limitations relevant to gait and upper limb function (SIS domains 6 & 7) at day 180. Beta coefficients are given with 95% confidence interval. *ICH volumes were transformed to natural logarithm to achieve normal distribution prior to analysis. IVH, intraventricular hemorrhage; ICH, intracerebral hemorrhage.

	**SIS domain 1 at day 180 (Motor impairment)**		**SIS domains 6&7 at day 180 (Activity limitation)**		**mRS day 180**	
	**β coefficient (95% CI)**	***p***	**β coefficient (95% CI)**	***p***	**β coefficient (95% CI)**	***p***
**Corticospinal tract involvement**						
CR	-18.91 (-46.20 to 8.38)	0.17	-65.88 (-121.0 to -10.77)	0.02	0.24 (-0.49 to 0.97)	0.25
PLIC	-3.04 (-20.02 to 13.94)	0.72	-32.23 (-66.52 to 2.06)	0.065	0.77 (-0.08 to 1.63)	0.08
Both	-17.39 (-32.98 to -1.80)	0.03	-52.78 (-84.25 to -21.30)	0.0013	0.72 (0.06 to 1.39)	0.03
Not involved	Ref		Ref		Ref	
**Sum of NIHSS motor domains (4 to 6) at baseline**	-4.06 (-5.75 to -2.37)	<0.0001	-7.37 (-10.79 to -3.945)	<0.0001	0.14 (0.07 to 0.21)	0.0002
**Age** (years)	-0.40 (-0.85 to 0.05)	0.083	-1.41 (-2.32 to -0.50)	0.003	0.03 (0.01 to 0.05)	0.003
**Sex** (Male)	7.51 (-3.73 to 18.75)	0.19	1.75 (-20.95 to 24.46)	0.88	0.1 (-0.41 to 0.61)	0.69
**IVH** (ml)	0.19 (-0.43 to 0.80)	0.55	0.48 (-0.77 to 1.72)	0.45	-0.02 (-0.05 to 0.01)	0.23
**ICH volume*** (ml)	-4.87 (-8.45 to 18.20)	0.47	-9.04 (-35.94 to 17.86)	0.51	0.35 (-0.24 to 0.94)	0.25
**Randomization to surgery**	-10.31 (-21.53 to 0.91)	0.07	-26.99 (-49.65 to -4.34)	0.02	-0.09 (-0.06 to 0.04)	0.74

Supplementary Table S5: Association between corticospinal tract involvement by novice observer SC and patient reported motor impairment and activity limitation. Results of multifactorial linear regression models to determine associations between corticospinal tract involvement classification by novice observer SC and patient reported motor impairment (Stroke Impact Scale [SIS] domain 1) and activity limitations relevant to gait and upper limb function (SIS domains 6 & 7) at day 180. Beta coefficients are given with 95% confidence interval. *ICH volumes were transformed to natural logarithm to achieve normal distribution prior to analysis. IVH, intraventricular hemorrhage; ICH, intracerebral hemorrhage.

	**SIS domain 1 at day 180 (Motor impairment)**		**SIS domains 6&7 at day 180 (Activity limitation)**		**mRS day 180**	
	**β coefficient (95% CI)**	***p***	**β coefficient (95% CI)**	***p***	**β coefficient (95% CI)**	***p***
**Corticospinal tract involvement**						
CR	-19.76 (-35.73 to -3.78)	0.016	-47.70 (-81.94 to -13.47)	0.007	0.95 (-0.27 to 2.18)	0.13
PLIC	-12.71 (-33.67 to -8.26)	0.23	-32.20 (-77.12 to 12.72)	0.16	0.27 (-0.52 to 1.05)	0.50
Both	-26.65 (-41.08 to -12.23)	0.0004	-50.63 (-81.54 to -19.72)	0.0017	0.87 (0.18 to 1.57)	0.01
Not involved	Ref		Ref		Ref	
**Sum of NIHSS motor domains (4 to 6) at baseline**	-3.88 (-5.53 to -2.23)	<0.0001	-7.26 (-10.80 to -3.72)	0.0001	0.13 (0.06 to 0.20)	0.0007
**Age** (years)	-0.40 (-0.86 to 0.06)	0.09	-1.30 (-2.29 to -0.32)	0.01	0.03 (0.01 to 0.05)	0.002
**Sex** (Male)	8.00 (-3.37 to 19.34)	0.17	3.58 (-20.75 to 27.90)	0.77	-0.02 (-0.52 to 0.48)	0.92
**IVH** (ml)	0.25 (-0.38 to 0.87)	0.43	0.51 (-0.83 to 1.85)	0.45	-0.01 (-0.04 to 0.02)	0.36
**ICH volume*** (ml)	14.95 (1.29 to 28.61)	0.033	11.80 (17.46 to 41.07)	0.42	0.44 (-0.13 to 1.01)	0.13
**Randomization to surgery**	-11.29 (-22.44 to -0.14)	0.047	-30.17 (-54.06 to -6.28)	0.014	-0.17 (-0.68 to 0.33)	0.50

Supplementary Figures

Supplementary Figure S1: Flow diagram of inclusion criteria and description of missing data. A diagram showing how participants for this study were selected, and a detailed description of missing data for each outcome variable included in the statistical analysis.

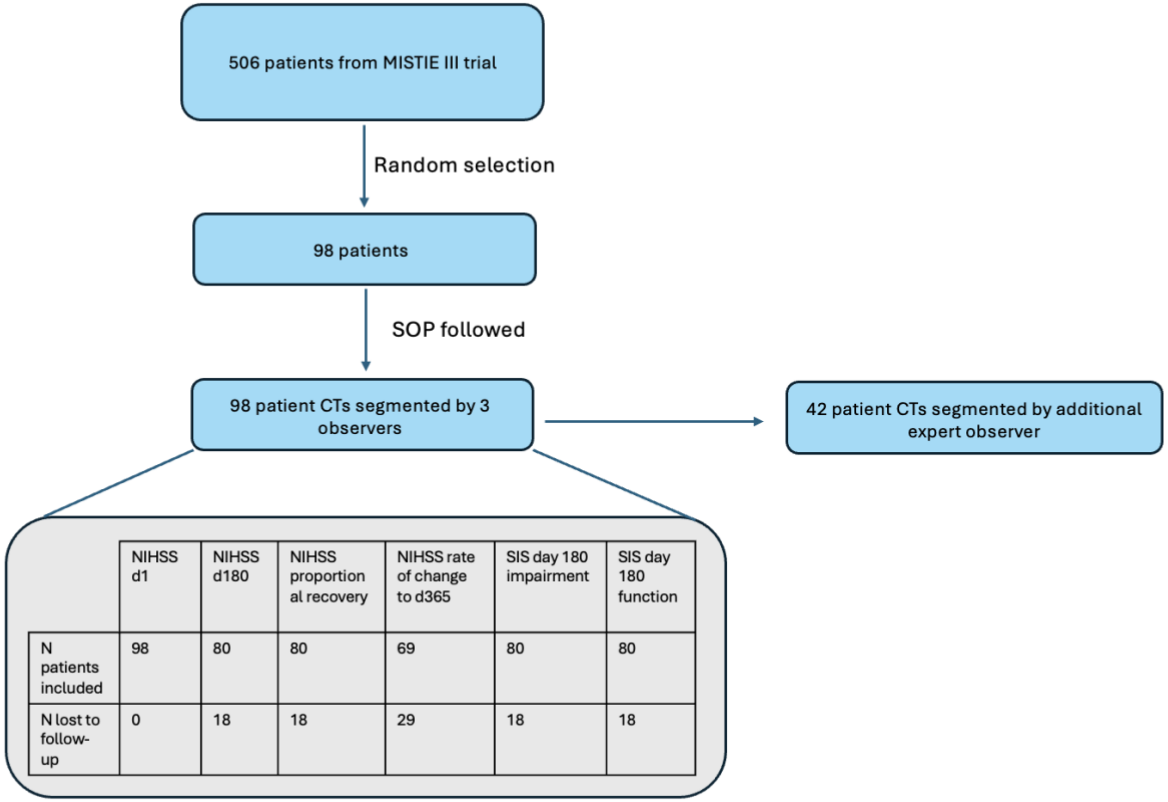

	NIHSS d1	NIHSS d180	NIHSS proportional recovery	NIHSS rate of change to d365	SIS day 180 impairment	SIS day 180 function
N patients included	98	80	80	69	80	80
N lost to follow-up	0	18	18	29	18	18

Data missingness for each outcome variable

VISTA – ICH Database Scan Analysis Guide

1. **Classify location:** Review multiple axial slices to visualise the location and spread of ICH. Other imaging planes may also be helpful. Based on this, Classify the site of ICH as DEEP (Lentiform, Caudate, Thalamic, Internal capsule), LOBAR (Frontal, Parietal, Temporal, Occipital, Insular), INFRATENTORIAL (Brainstem, Cerebellum) or UNCERTAIN using the definitions in appendix.
2. **Classify corona radiata involvement:** Classify whether the corticospinal tract at the level of the corona radiata is involved, using the most rostral slice with the septum pellucidum visible (see Fig 1). Divide the lateral ventricle in thirds along the anterior-posterior axis. Assume the corticospinal tract is located lateral to the middle third of the ventricle (Fig 1).
    a. **Draw** an approximation of the region in Fig 1 using the 'pencil' tool to approximate a rectangle. Horizontal lines should be 1cm and the vertical line the middle third of the lateral ventricle wall.
    b. **Define** this region as:
        i. 'Not involved' – no blood involving the region
        ii. 'Partially involved' - haematoma involves some but not all this region iii. 'Completely involved' – this region cannot be seen due to the haematoma

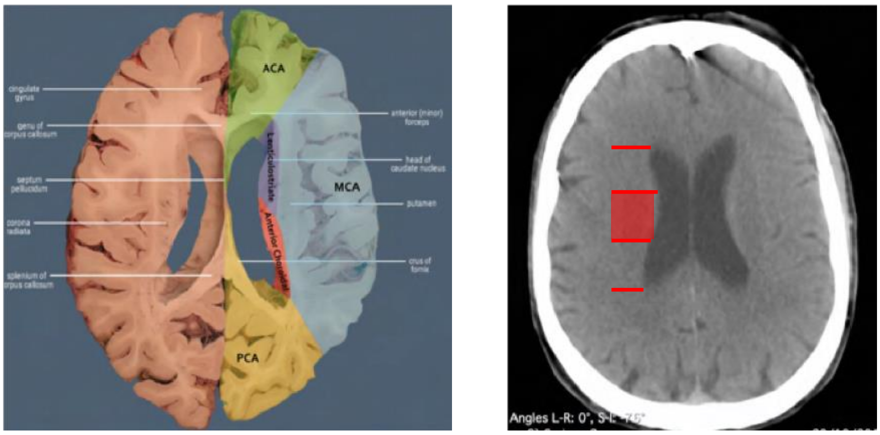

Fig 1: CT at level of corona radiata. An approximation of the region to define the involvement of the corticospinal tract at this level is demonstrated, as per step 2 (shaded red).

    c. **Measure** haematoma distance from midpoint of corticospinal tract in corona radiata:
        i. Find the most rostral slice with the septum pellucidum visible (as per Fig 1)
        ii. Drop a measurement point halfway along the lateral wall of the lateral ventricle iii. Measure the shortest 3D distance to haematoma border

iv. Define as ‘0’ if the point defined in step ‘b’ is involved in the haematoma 3. **Classify posterior limb of the internal capsule (PLIC) involvement**: using all slices where the lentiform nucleus and thalamus are clearly visible, ipsilateral and/or contralateral to the ICH (Fig 2).

a. **Draw** around the PLIC (as in Fig 2) using the ‘pencil’ or ‘closed polygon’ tool on every slice that it is visible.

b. **Define** as:

i. ‘Not involved’ – all of the ipsilateral PLIC can be seen

ii. ‘Partially involved’ - some ipsilateral PLIC can still be seen, but haematoma involves some of the PLIC. If partially involved, determine which anatomical area of the PLIC is affected, whether, anterior, middle and/or posterior.

iii. ‘Completely involved’ - no ipsilateral PLIC can be seen due to the haematoma

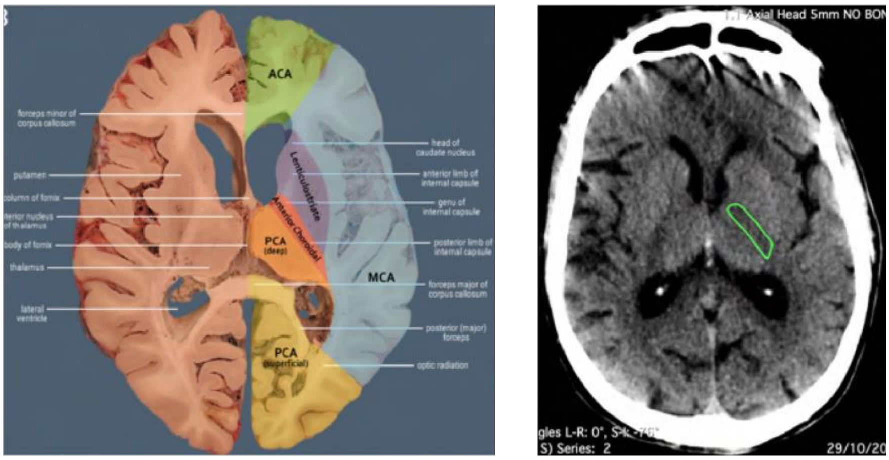

Fig 2: Axial slices at the level of the basal ganglia and thalamus, with the left PLIC outlined in green on the example CT scan.

c. **Measure** the shortest distance from the PLIC ROIs drawn in previous step and the haematoma, measuring in 3D if required.

4. **Classify anterior limb of the internal capsule (ALIC) involvement**: using all slices where the lentiform nucleus and thalamus are clearly visible, ipsilateral and/or contralateral to the ICH (Fig 3). Define as:

a. ‘Not involved’ – all of the ipsilateral ALIC can be seen

b. ‘Partially involved’ - some ipsilateral ALIC can still be seen, but haematoma involves some of the PLIC. If partially involved, determine which anatomical area of the PLIC is affected, whether, anterior, middle and/or posterior.

c. ‘Completely involved’ - no ipsilateral ALIC can be seen due to the haematoma. Define measurement as ‘0’ for step 5 for these cases.

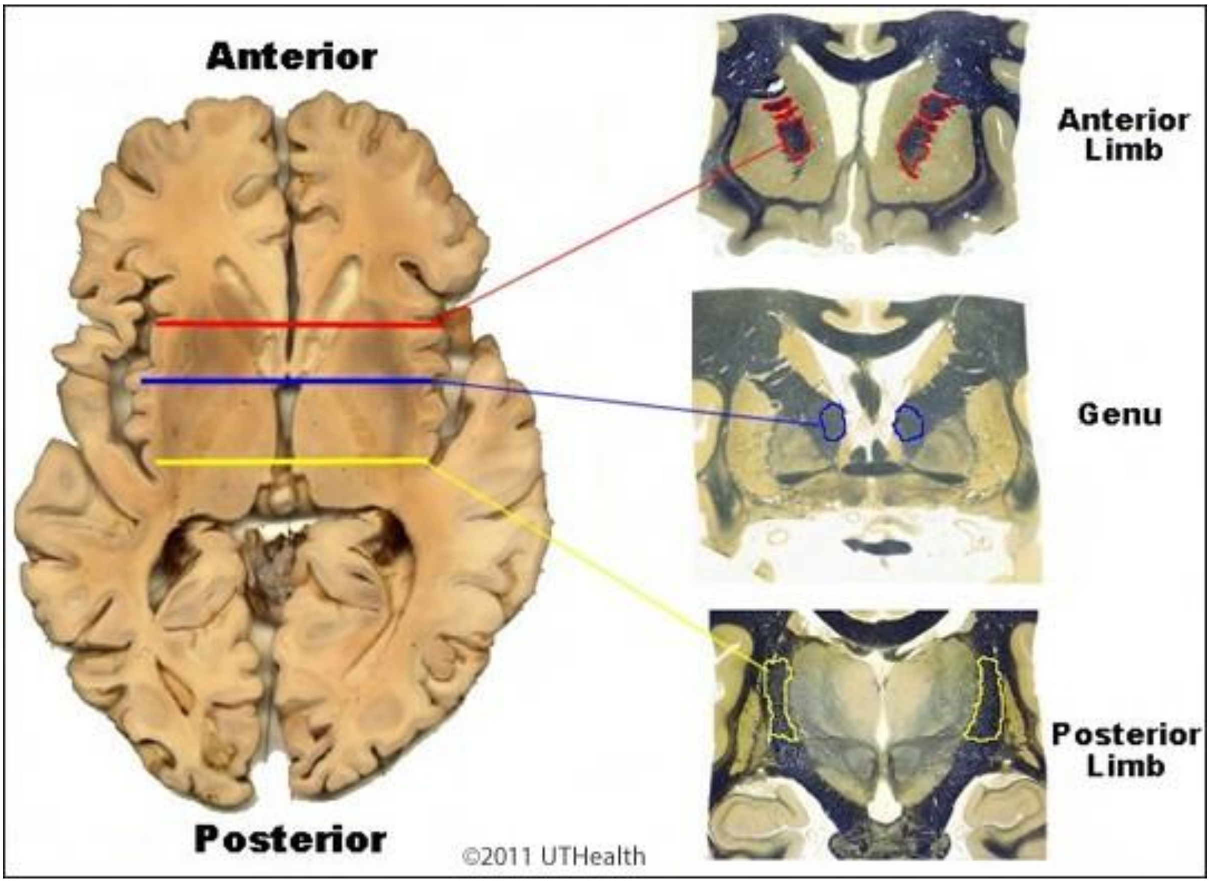

Fig 3: Axial slices at the level of the basal ganglia and thalamus, with all three limbs of the internal capsule highlighted in red, blue and yellow respectively(*Neuroanatomy Online: Lab 2 - Internal Organization of the Brain - Internal Capsule*, 01/04/2021).

5. **Measure** the shortest distance from the ALIC ROIs drawn in previous step and the haematoma, measuring in 3D if required.
6. Save all regions of interest (ROI) on completion.

Appendix: Defining location

- **Lobar ICH:** the main bulk and the presumed epicentre of the haematoma is located in the cerebral cortex or at the junction of the cortex and white matter (*including subcortical white matter*) and does not extend into the subcortical gray matter structures such as the basal ganglia or thalamus. Lobar ICH may be further subdivided according to lobes:

- Frontal
- Parietal
- Temporal
- Occipital
- Insular

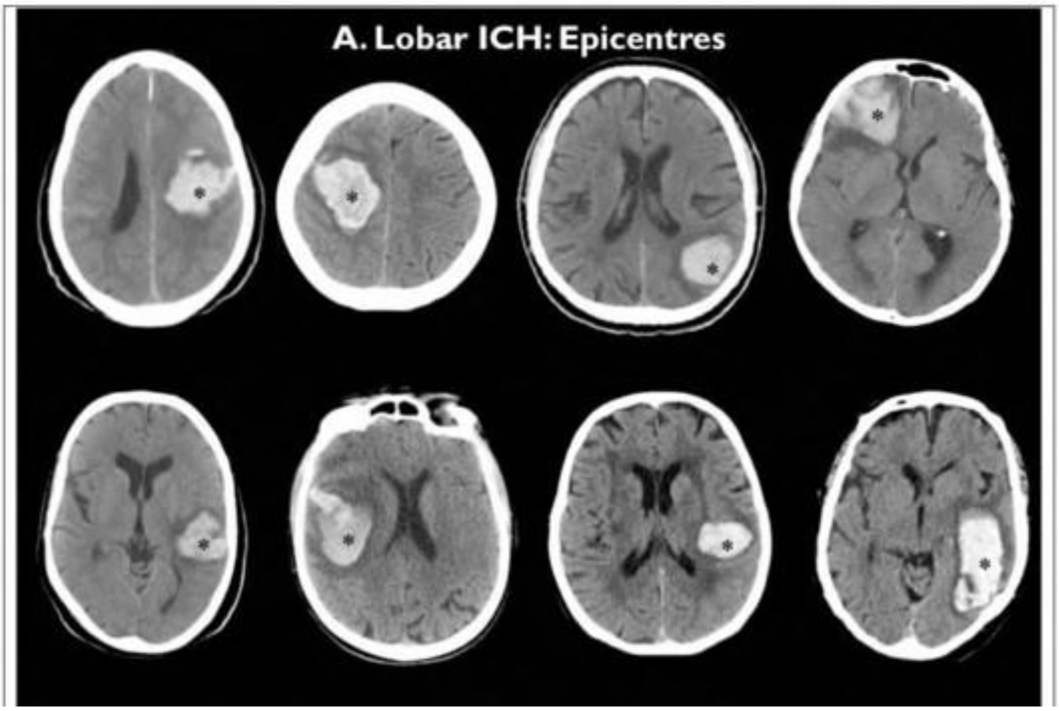

Fig : Classification examples for location of ICH (Charidimou *et al.*, 2017)

- **Infratentorial:** the main bulk of the hematoma is located below the tentorium cerebelli; the region contains the cerebellum. The infratentorial subsection can be further subdivided into:
  - Brainstem
  - Cerebellum.
- **Deep Brain structures:** the main bulk of the haematoma located in the basal ganglia, thalamus, brainstem and usually does not extend into cerebral cortical grey matter. Deep brain ICH may be further divided as:
  - Lentiform
  - Caudate
  - Thalamus
  - Internal Capsule

  → Anterior limb

  → Genu

→ Posterior limb

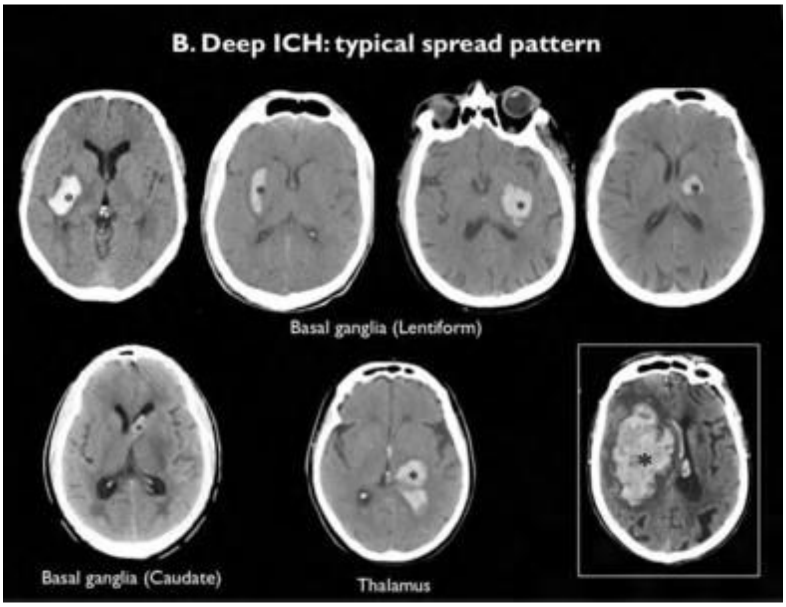

Fig 4: Classification examples for location of ICH (Charidimou *et al.*, 2017)

- **Uncertain:** here the ICH is difficult to distinguish visually between lobar and non-lobar origin (e.g., the ICH is too large and extends into both lobar and non-lobar areas), the location should be recorded as "Uncertain".
